# Teaching about antibiotic resistance to a broad audience: a multidisciplinary approach

**DOI:** 10.1093/femsle/fnaa111

**Published:** 2020-06-30

**Authors:** Kristian Kvint, Martin Palm, Anne Farewell

**Affiliations:** Department of Infectious Diseases, Institute of Biomedicine, The Sahlgrenska Academy at University of Gothenburg, Guldhedsdsgatan 10A, SE-413 46, Sweden; Centre for Antibiotic Resistance Research (CARe), University of Gothenburg, Guldhedsgatan 10A, SE-413 46 Gothenburg, Sweden; Department of Chemistry and Molecular Biology, University of Gothenburg, Medicinaregatan 9E, SE-405 30, Sweden; Centre for Antibiotic Resistance Research (CARe), University of Gothenburg, Guldhedsgatan 10A, SE-413 46 Gothenburg, Sweden; Department of Chemistry and Molecular Biology, University of Gothenburg, Medicinaregatan 9E, SE-405 30, Sweden; Centre for Antibiotic Resistance Research (CARe), University of Gothenburg, Guldhedsgatan 10A, SE-413 46 Gothenburg, Sweden

**Keywords:** antibiotic resistance, education, online course, multidisciplinary, bacteria, antibiotic

## Abstract

Education for the general public about antibiotic resistance is advocated as a key component of our response to this crisis. Since this is a multidisciplinary problem encompassing natural, medical and social sciences, it is an educational challenge as both students and lecturers will have vastly different backgrounds in the topics. Here we describe an online multidisciplinary course on antibiotic resistance spanning topics as diverse as chemistry and practical philosophy. The target group was any post-secondary school student and the participating students had different occupations and educational experience. Although as many as 38% of the students were currently studying natural sciences at university, the course included a diverse group with medical professionals (16%) and teachers (6%) making up a significant fraction of the class. The outcomes based on examination and the course evaluations were very positive and we have indications that the information students gained from this course has been spread to others. Unlike other online courses addressing antibiotic resistance, this course is both accessible to a wide range of students and covers a broad range of topics. We advocate courses like ours as an effective tool in educating the public about this crisis.

## Teaser

This free online course teaches about antibiotic resistance to a broad audience and addresses the issue from many diverse perspectives including biology, chemistry, politics and economics.

## INTRODUCTION

Education about the problem of antibiotic resistance is a cornerstone of the WHO global action plan on antibacterial resistance, as it is in most national action plans (World Health Organization 2015, 2020). Overuse and misuse of antibiotics in medicine and food production has contributed greatly to the current crisis: e.g. antibiotic use (dose/person) varies more than 10-fold in different regions of the world (World Health Organization 2018). The reasons for overuse and misuse of antibiotics are complex, reflecting economic, political and psychological factors (Hoffman *et al*. 2015). Further, the difficulties of new drug development have resulted in few new antibiotics being approved in recent years and few pharmaceutical companies working on antibiotic development (Nathan 2004). The One Health perspective (Hernando-Amado *et al*. 2019) is widely advocated to consider all sources of antibiotic resistance development: humans, animals and the environment. This perspective brings together knowledge from medical doctors, veterinarians, and food producers as well as environmental sciences.

Thus, the problem of antibiotic resistance is highly complex: biological, chemical, political, ethical, ecological, medical, economic and psychological mechanisms have been pin-pointed as contributing to the problem. A multi-disciplinary approach is required both in our response to the issue and education about it. In addition, university education encompassing multi-disciplinary fields is often a challenge as most lecturers are specialized in a single scientific discipline. This creates an educational challenge: how can we educate people with vastly varying backgrounds about the current problem?

In this paper, we describe a multidisciplinary course on antibiotic resistance developed at the Centre for Antibiotic Resistance Research (CARe) at the University of Gothenburg (GU), Sweden. The course was aimed to educate and improve awareness and understanding of antibiotic resistance for any post-secondary school student but was especially targeted to high-school teachers and current university students in any discipline. We focused on this target group as, especially for high-school teachers, we would expect their knowledge to continue to spread to a wider audience. The lecturers in the course originated from five different faculties at GU, thus covering the multi-disciplinary breadth required for understanding the comprehensive issues of antibiotic resistance.  Because we expected a diverse student group the course started with very basic lectures on bacteria and antibiotics but quickly progressed to current research in the different fields on the topic. The course was offered both as an evening course and as a purely online course. This paper describes the administration, content and evaluation of the online course. Although other online courses on this topic exist, they do not target this audience with the same breadth and depth of topics. Based on our experience, we strongly suggest that courses like this should be made available to a wider audience to educate the general public about the issues concerning this global crisis.

## MATERIALS AND METHODS

### Course administration, structure and examination

The online course ‘The Problem of Antibiotic Resistance’ (3 ECTS, equivalent to 1.5 course credits in the US system) was open to anyone with the basic prerequisites to study at a university in Sweden in any subject. Students were required to register through the Swedish higher education portal (*www.universityadmissions.se*). University education is free in Sweden and is also offered free to any EU citizen; students outside the EU would be charged tuition.

The course was run using a learning management system Canvas in 2019 (Instructure Inc.) and GUL in 2017–2018 (Ping Pong AB). All material and communication were done through this platform. A module or page was created for each lecture hour and contained the embedded videos (hosted on YouTube), a quiz for the module and slides from each lecture, as well as links to further information or background information. A discussion forum was available for students to ask questions. The videos and other material were made available each week (two lectures/week) for 6 weeks. The students were able to complete the quizzes at their own pace over the 10-week semester.

Examination of the course was done using online quizzes. Students needed to successfully pass a 3-question multiple choice quiz (expanded to five questions in 2019) after watching each lecture. Quizzes are available at *antibiotic-resistance.org*. All quizzes were automatically graded on our course management system. Overall, the students needed an average grade of 60% to pass the course and the course was graded pass/fail. There were only 10 graded quizzes for the 12 lectures (excluding quizzes for Ethics (lecture 8) and Current Research (lecture 12)). In the Swedish system, students have the right to reexamination and new quizzes were provided for those who did not successfully pass the course the first time.

### Video production

Lectures given to an evening class of ∼25 students in 2017 and again in 2019 were recorded using an in-built video recording system in the classroom in 2017 or with an improved system (in 2019) using an SLR camera on a tripod and a lapel microphone connected to a laptop computer and recorded using Camtasia v. 8 (TechSmith Corp., Okemos, MI, USA). Only the lecturer was recorded. Later, using Camtasia, the slides were synched with the lecturers’ presentations, students' oral questions were either edited out completely or placed as text in the video and highlights and arrows were added to emphasize parts of the slides as needed. Finally, the video was produced in Camtasia and uploaded onto YouTube (YouTube 2020). Two versions of each lecture were created: one full-length 40–45-minute video and several 5–20 min videos. Students could choose which versions they preferred.

### Course evaluation surveys and analysis

Course evaluations were administered online at the end of each course. The course evaluation structure and questions (Supplemental text) were standardized throughout the Faculty of Sciences in 2018 and have been tested extensively. Analysis of the text-based answers were done by identifying four categories of interest and two independent researchers scored the responses. The categories were: (1) positive or negative about the course in general (2) thought the multidisciplinary nature of the course was positive/negative (3) usefulness of the course in the future and (4) that though they had knowledge in one area of the course, the other topics were appreciated. Note the number of responses in the 2019 course evaluation were much lower than in 2018 (38 vs. 157) due to technical problems at our university with a new course evaluation system thus these were only minimally analysed. The follow-up survey was sent to the students in the 2018 course using the Sunet (Swedish University computer Network) platform.

### Ethics statement

Anonymized course evaluation results and survey summaries are public records and considered part of the course report. In the after-course follow-up survey, students were informed of the reason for the survey and told that their anonymous comments could be included in a publication. Participation for all surveys was entirely voluntary and the anonymity was guaranteed using the Sunet survey platform (2019 and follow-up survey) or GUL (2018) which hides all identifying information from the researcher. Students in the evening course were aware that the lecturer was being recorded but that their voices (asking questions) would be removed from the videos before publication.

## RESULTS

### Course design

The course ‘The Problem of Antibiotic Resistance’ was created as part of our work to increase basic knowledge of the issues surrounding antibiotic resistance for the general public. This university level course was open to anyone who had basic prerequisites to enter university in any field and registered for the three ECTS course. Thus, an important consideration in the design of this course was to both make sure there was enough background information given so that everyone could understand (i.e. basic cell biology for those who had not studied biology) but yet remain engaging enough for those who had strong backgrounds in these topics. This was accomplished by choosing to teach at a first-year university/upper secondary school level for all background information but yet leading them through to modern research topics. The background material was highly focused on information needed to understand the basic concepts as well as higher level research topics. Thus, the course was broad in the sense that students were exposed to many disciplines but was very narrowly focused on topics needed to understand antibiotic resistance (ABR).

We aimed to make the course multidisciplinary, covering topics ranging from philosophy to molecular biology. All teachers aimed to make their lectures understandable to people with little knowledge of the topic except that which was covered in the previous lectures of the course. The course was designed to take place over 6 weeks with two 45-minute video lectures per week. The topics and learning objectives for each lecture are described in Table 1. The lectures were recorded in an evening course and subsequently edited for clarity (see Materials and Methods) and posted for the online course. Students were further provided with the slides from each lecture as well as links to further information on each topic. The course required that the students successfully pass a multiple-choice quiz after watching each lecture (see Materials and Methods).

**Table 1.** Topics and Learning Objectives for each of the lectures in the course.

Topic	Field of study	Students can describe or explain:
The Problem of Antibiotic Resistance	General	- the scope and seriousness of the problem both nationally and worldwide
		- the main factors contributing to the problem of ABR
Introduction to the Bacteria and Cell Biology	Molecular Biology	- what bacteria are, how they grow and divide and the basic bacterial cell structure
		- what a gene is and how a mutation can change protein structure
Antibiotics and Antibiotic Resistance	Molecular Biology/Chemistry	- mechanism of action of several classes of antibiotics including beta-lactams, ciprofloxacin and ribosome-targeting antibiotics
		- mechanisms of resistance including efflux pumps, target modification and drug inactivation
Development of Antibiotic resistance	Molecular Biology	- how mutations can occur and lead to ABR
		- how genes can spread through horizontal gene transfer in bacteria including conjugation, transduction and transformation
Non-human Use of Antibiotics	Environmental Science	- the One Health Perspective
		- how misuse of antibiotics in food production can impact the problem of ABR in humans
Environmental Dimensions of Antibiotic Resistance	Environmental Science	- how environmental pollution of antibiotics from manufacturing, farming and human use impacts the problem of ABR
		- how environmental pollution impacts transmission and evolution of resistance
Clinical Consequences of Antibiotic Resistance	Medicine	- considerations for a clinician with regard to ABR
		- need for faster diagnostics
		- increased costs to patients with increasing ABR
Ethics of Antibiotic Use	Philosophy	- ethical dilemmas which arise in the response to ABR
		- how different roles and perspectives can lead to social conflicts in our response to ABR
		- collective action problems
Medicinal Chemistry and Development of New Antibiotics	Chemistry	- the importance of pharmacokinetics
		- the concept of structure-activity relationships
		- approaches to new antibiotic drug development
The Economics of New Antibiotics	Economics	- economic cost of ABR
		- incentives vs. bans to limit inappropriate antibiotic use
The Politics of Antibiotic Use and Misuse	Political Science	- how political interventions can aid or hinder in the response to the ABR crisis
		- regulated vs. voluntary collective action approaches
Current Research	General	- some of the current approaches to the problem of ABR

### Course overview

The course was developed and first run in 2017. It was offered in the evening in 2017 and 2019 and recorded for the online course each time. The online course attracted a large number of students (193, 342 and 254 students in 2017, 2018 and 2019, respectively). A companion lab course was also offered (5 days full time) and 24 students have taken and passed this course to date. Information about the lab course is available online (*antibiotic-resistance.org/lab-course*). In this paper, we focus primarily on the online course from 2018 to 2019.

### The student population

**Figure 1.**
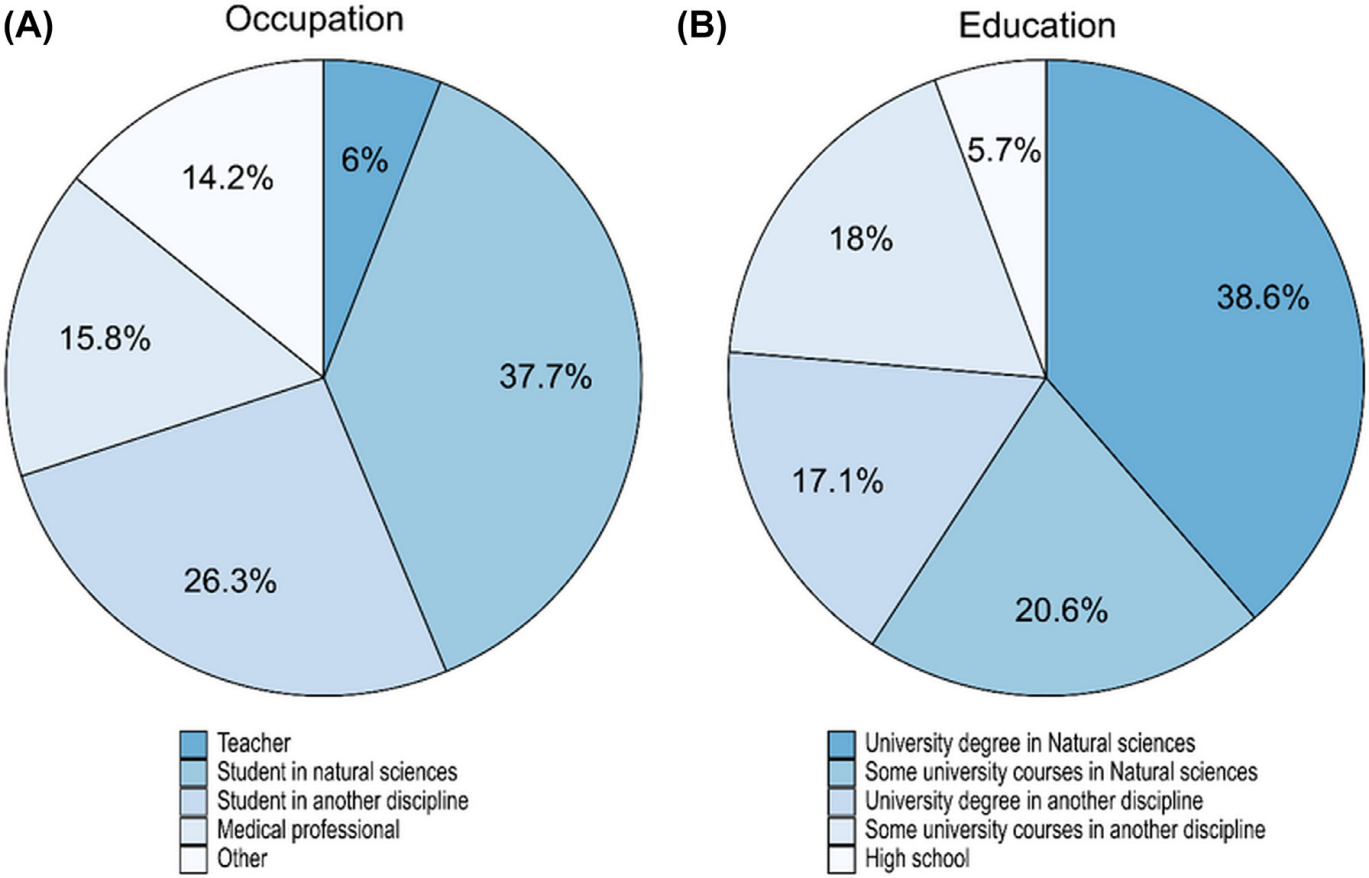
Student Population. A survey was done at the start of the 2018 and 2019 online course asking students about their background: current occupation is plotted in **(A)** and highest obtained education level is in **(B)**. 52% (316) of the registered students responded to this survey.

Pre-course surveys were done in the online courses in 2018 and 2019 (317 (53%) students responded) to identify our students’ backgrounds. The student composition of the two courses is described in Fig. 1 with 41% students having had no university level natural sciences courses and the remainder either studying or had studied at least some natural sciences. Overall, the majority of students in the course were full-time university students. We purposely advertised this course as a continuing education course for teachers and unsurprisingly 6% of the students were active secondary school teachers. Amongst those with an ‘other’ occupation, many (16%) were medical professionals (doctors, nurses, dentists, laboratory technicians, veterinarians and pharmacists) but students’ occupations also included IT consultants, political scientists, engineers, lawyers, farmers and bankers. Thus, our student group was highly diverse with respect to occupation and educational background.

### Course outcomes

The main aim of this course was to increase knowledge and understanding of the issues regarding ABR, not to prepare people for future work in the field, thus the bar for passing this course, *if they watched all the videos*, was set rather low. Thus, a large percentage of the students who actually participated in the course successfully passed. Overall, 74% of the students who initially registered completed the course (582 students total 2017–2019). It should be noted that since Sweden does not have tuition fees for EU citizens, there was no financial consequence for those who registered but did not complete the course. The majority of students who did not complete the course either watched 0 or 1 video without continuing.

**Figure 2.**
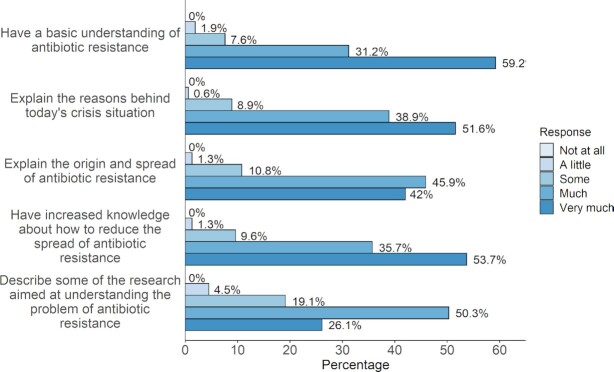
Course evaluations. Students were asked how well they had learned each of the learning objectives listed. A total of 158 students (46%) answered the survey that was done after the 2018 course.

The student evaluations of the course were very positive. The results of part of the 2018 online course evaluations (157 responses) are summarized in Fig. 2. The majority of students reported they learned the main course objectives either well or very well (87% average for all five learning objectives). It was clear however, that the students had more confidence in their knowledge of more basic learning objectives (‘have a basic understanding of antibiotic resistance’) than more difficult objectives (‘describe some of the research aimed at understanding the problem of antibiotic resistance’) with 59% and 26%, respectively, reporting they learned this very well. In the open format text boxes, students’ comments (95 comments received) were overall very positive (‘good’ course: 63 comments, 68%; ‘bad’ course: 2 comments, 2%). In particular, many of the students (20 comments, 22%) commented that they appreciated the multidisciplinary aspects of the course. Many commented that though they had rather deep knowledge about one of the subject areas (e.g. biology), they appreciated the breadth of knowledge they gained from other disciplines (9 comments, 10%). Further, a number of medical professionals also noted that they thought this course would help them further in their work (6 comments).

Finally, we have indications that the information students gained from this course is spread to others. A year after the course, we sent a survey to the students we were able to contact from the 2018 online course and asked if they had spread or used the information obtained in the course. Although only 32 students answered, the majority (78%) who answered said yes, including high school teachers who taught about the topic, medical professionals who used it in their work and students who spread the information on social media and to family and friends. Notably, we have also heard positive comments from professionals (e.g. lawyers and administrators) who work closely associated to ABR topics but have not had the opportunity before to understand the work being done in their organization.

### Conclusions and future perspectives

The creation of the Centre for Antibiotic Resistance Research (CARe) at our university gave us a unique opportunity to organize a multi-disciplinary course covering a wide range of topics related to the problem of antibiotic resistance. However, it should be reasonably easy for other universities to organize a course along these lines using the template we have created. Further, the entire course including quizzes is available at *antibiotic-resistance.org* and the videos are available both at the CARe website and YouTube (CARe 2020; YouTube 2020). They are available to use with a creative commons license. The videos have been edited into short segments thus allowing their individual use in other contexts (e.g. a secondary school class) and are available at the same sites.

An open question concerns the most effective pedagogy for us to meet our aims of increasing awareness and knowledge about the problem of antibiotic resistance. Numerous online courses and resources have been developed for medical students and practicing health care professionals (e.g. Sneddon *et al*. 2018; Coursera 2020; Tsopra *et al*. 2020), and these generally have been found to be effective in changing prescribing habits at least in the short term (Lee *et al*. 2015 ). In addition to resources for health care workers, some educational resources target school children (e.g. McNulty *et al*. 2011), high school or university students (Hernandez *et al*. 2018; Small World Initiative 2020) and outreach programs, such as educational pamphlets (US FDA 2020; Public Health England 2020) or events (Redfern *et al*. 2018). These have been widely used to varying degrees of success, however, a systematic review of the available literature concludes that there is currently too little data available to make a firm conclusion as to the best way to change long-term antibiotic behaviors of the general public (Price *et al*. 2018). If possible, it would be informative to re-survey our students about antibiotic knowledge and behavior after a period of years to determine if we have had a long-term impact. It should be noted however, that this particular course would not substitute for other outreach activities and materials (e.g. pamphlets or events) as it requires a much larger commitment of time for the students than other approaches. Thus, it should be considered another tool at our disposal.

Future work with this course will be to increase the available feedback to students in the online course using the discussion forums, video chats and embedded quizzes. Further, we think new more advanced online course, building on this one, could cover some topics in more detail (e.g. current research in the field) and topics not addressed such as vaccine development, immunology and pathogenesis. We expect that the continuation course could strengthen the students’ knowledge of topics such as current research which our students were less confident about (Fig. 2). Furthermore, both the current course and the planned one are relevant not only to the ABR crisis but overlaps significantly with topics relevant to the Covid-19 pandemic (e.g. resistance, vaccine development and social interventions).

Unlike other online courses about ABR which most commonly target health professionals, our course is much more accessible to a broad audience and covers topics not often included in such a course (i.e. ethics and social sciences). In conclusion, we have created an accessible course for a wide audience which will contribute to the global education on antibiotic resistance.

## Supplementary Material

fnaa111_Supplemental_File
