# Supplementary material for: Teaching about antibiotic resistance to a broad audience: a multidisciplinary approach
Source: FEMS Microbiol Lett. 2020 Jun 30;367(14):fnaa111. doi: 10.1093/femsle/fnaa111 (PMC8962684; doi:10.1093/femsle/fnaa111)
Supplement: fnaa111_Supplemental_File [file fnaa111_supplemental_file.docx]

**Supplemental Text**

Student Course Evaluation 2018

1. Do you think you learned to:

|  | 1 Not at all | 2 A little | 3 Some | 4 Much | 5 Very much |
| --- | --- | --- | --- | --- | --- |
| Have a basic understanding of antibiotic resistance | 🞏 | 🞏 | 🞏 | 🞏 | 🞏 |
| Explain the origins and spread of antibiotic resistance | 🞏 | 🞏 | 🞏 | 🞏 | 🞏 |
| Explain the causes of the current crisis | 🞏 | 🞏 | 🞏 | 🞏 | 🞏 |
| Have increased knowledge about how to reduce the spread of antibiotic resistance | 🞏 | 🞏 | 🞏 | 🞏 | 🞏 |
| Describe some of the current research approaches to the problem | 🞏 | 🞏 | 🞏 | 🞏 | 🞏 |

2. Was the course structured such that you felt you had sufficient support to learn the topics?

(Yes/No) If not, please explain.

{Text Box}

3. How much did these tools/teaching methods help in your learning?

|  | Don’t know | 1 Not at all | 2 A little | 3 Some | 4 Much | 5 Very much |
| --- | --- | --- | --- | --- | --- | --- |
| Lectures | 🞏 | 🞏 | 🞏 | 🞏 | 🞏 | 🞏 |
| Real Life Examples | 🞏 | 🞏 | 🞏 | 🞏 | 🞏 | 🞏 |

4. Was information sufficient, easily available and provided in a timely manner?

Yes/no. If not, please explain

{Text Box}

5. What was your overall impression of the course?

|  | Don’t know | 1 Disagree | 2 Mostly disagree | 3 Neither agree/disagree | 4 Mostly agree | 5 Agree |
| --- | --- | --- | --- | --- | --- | --- |
| The course was difficult | 🞏 | 🞏 | 🞏 | 🞏 | 🞏 | 🞏 |
| I became very motivated in this course | 🞏 | 🞏 | 🞏 | 🞏 | 🞏 | 🞏 |
| The course was interesting | 🞏 | 🞏 | 🞏 | 🞏 | 🞏 | 🞏 |
| The quality of teaching was very good | 🞏 | 🞏 | 🞏 | 🞏 | 🞏 | 🞏 |

6. The course's organisation should not discriminate against anyone in any way, for example, by gender, ethnicity, age or family situation. If the course has failed in this objective, describe how.

{Text Box}

7. Is there anything else you would like to add? For example, what was particularly good in this course, what would you like to change in this course, was it at the right level for you?

{Text Box}
